# Low voltage optical fiber positioner robot based on minimum inductance hollow cup motors

**DOI:** 10.1038/s41598-022-06214-7

**Published:** 2022-02-17

**Authors:** Shaoxiong Guo, Chao Zhai, Songlin Bi

**Affiliations:** 1 Department of Precision Machinery and Precision Instrumentation, University of Science and Technology of China, No.96, JinZhai Road Baohe District, Hefei, Anhui 230026 China; 2 Experiment Center of Engineering and Material Science, University of Science and Technology of China, No.96, JinZhai Road Baohe District, Hefei, Anhui 230026 China

**Keywords:** Electrical and electronic engineering, Mechanical engineering, Astronomy and astrophysics

## Abstract

With the further transformation of The Large Sky Area Multi-Object Fiber Spectroscopic Telescope, the new generation of fiber positioner robot chooses a 4 mm hollow cup motor with minimum phase inductance. Because the load of the fiber positioner robot is constant and the inertia of the motor is very small, an open loop positioning control method based on Space Vector Pulse Width Modulation is proposed, and the specific open loop parameters are directly tuned by relevant experimental strategies. The critical factors of the open loop driving mode are discussed in detail from four aspects: subdivision, fundamental frequency, wave generation mode and peak current. Based on the actual fiber positioner robot, the hardware driver and assessment platform are built. The positioning tests show that the method proposed is practical and effective, and meets the precision positioning demand of the new generation optical fiber positioner robot.

## Introduction

**Figure 1:**
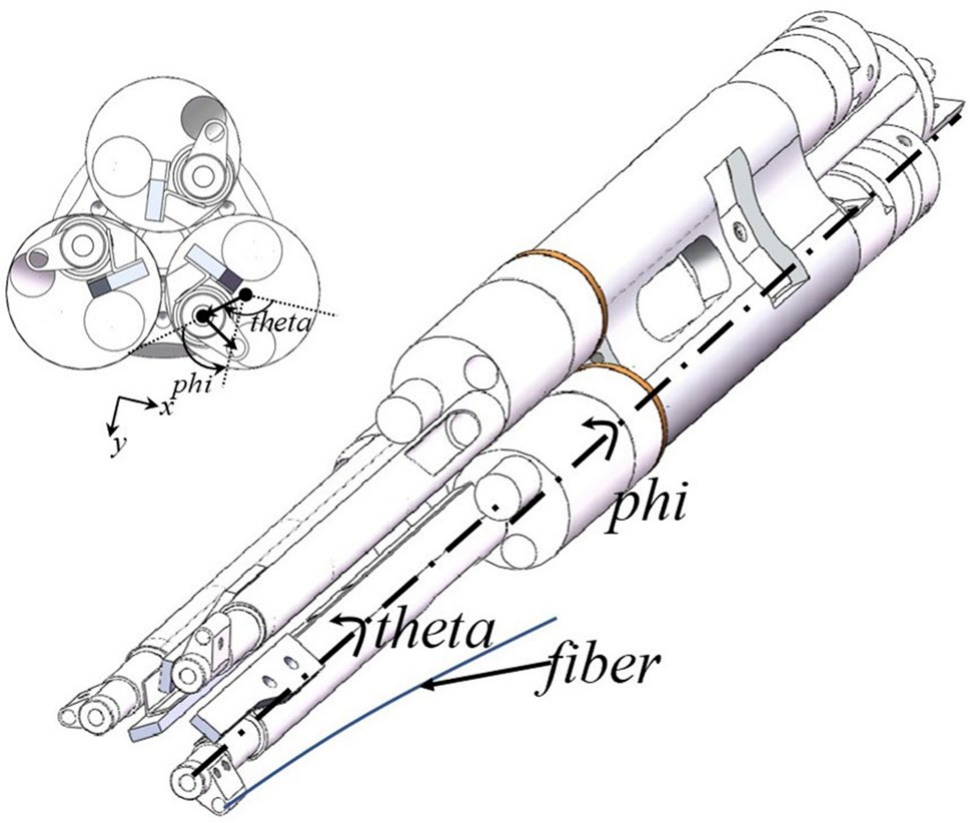
Each optical fiber positioning robot consists of two rotary axes (theta and phi), positioning the optical fiber on the focal panel in micrometer precison, and three such robots make a group.

LAMOST^1^ (The Large Sky Area Multi-Object Fiber Spectroscopic Telescope) is a Schmidt telescope for spectral measurement with large aperture and field of view, and a focal panel with 4000 optical fiber positioner robots is its most important part. With the further transformation of the astronomical telescope LAMOST project, the panel is designed to be smaller and the number of positioner robots increases to 5000, hence the mechanical design of fiber positioner robots (Fig. 1) used to place the optical fiber is more compact, mainly because of the physical size limitation, the miniature hollow cup motor (Table 1) as the driving unit of the mechanical structure has become the preferred scheme. At the same time, because the miniature hollow cup motor^2^ completely cancels the magnetic core medium in the stator, eliminating the eddy current loss and hysteresis core loss of the iron core, the energy loss is lower and the efficiency is higher^3^, which brings great improvement to the seeing of the whole telescope system^4^.

**Table 1:** Hollow cup motor parameter.

BMN04-8	Value	Unit
Pole pairs	2-pole	
Max sppilication speed	24200	rpm
r (Single phase resistance)	14.85	Ohm
Ls (Coil inductance)	37	uH
Ld (the inductance of d axis)	37	uH
Lq (the inductance of q axis)	37	uH
Ld/Lq ratio	1	
BEMF constant	0.059	mV/rpm
Inertia	0.00087	gcm$^2$

Throughout the whole field of theta-phi optical fiber positioners, the classical stepper motor^5^ is promising to precise positioning control, e.g. MEGARA^6^ and MOONS^7,8^ astronomical observation system. The stepper motor is robust and easy to drive because of its robust ablility of positioning control, which is also the positioning scheme used in the previous generation of LAMOST drive control system^1^. Hollow cup motor belongs to BLDC (Brushless Direct Current) motor, because of its superior speed performance in a wide range of speed regulation, it is widely used in many speed regulation applications.

But in the precision positioning control field, there are two closed loop control schemes for BLDC motor: sensored and sensorless.

- sensored closed loop control, the encoder is used^9,10^, based on FOC (Field-oriented control) or other control methods to form a position closed loop negative feedback control system to meet the demands of precision positioning. This is also the control scheme selected by SDSS-V Sloan Astronomical observation system^11^.
- sensorless closed loop control, divided into two categories according to the principle of detecting rotor position:

- The BEMF (back electromotive force) method^12,13^ uses BEMF model to estimate the rotor position; the theoretical application scenario is that rotor spins at medium or high speed, when there is a fair adequate BEMF to estimate the rotor position. But when motor is in zero speed or low speed, i.e., there is no BEMF or BEMF signal will be very weak, a series of rotor position estimation algorithms based on BEMF model, e.g. SMO^14–16^, Adaptive methods^17^, State Observer^18^ and AI (Artificial Intelligence) methods^19–21^, will not be applicable.
- The HFI (high-frequency injection) methods^22,23^ depends on the motor convex polarity model^24^ (decomposing the angle information between 0 to $180^\circ$) and the stator magnetic saturation model^25^ (distinguishing north or south pole information).

**Figure 2:**
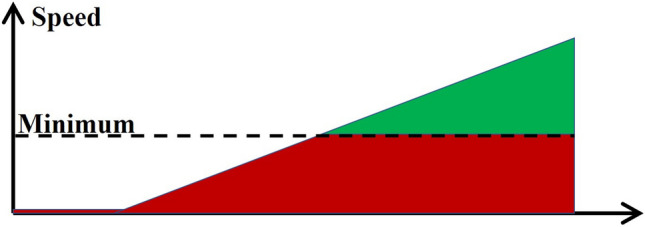
Red part represents low-speed, green high-speed.

The method based on the BEMF model is applicable to the green part of the Fig. 2. A special zero-speed phase and the low-speed range have to use HFI algorithm, which applied to the red part of the Fig. 2. Usually, the combination of the above two methods can complete the positioning control in the full speed range.

However, the selected miniature 4 mm hollow cup motor does not have a 4 mm encoder matching its size as the position feedback unit in a closed loop position control system, and the inner rotor of the motor is composed of a whole magnet without convex polarity. Additionally, its ironless windings has no characteristic of inductance saturation.

On the other hand, the carrier frequency of PWM (Pulse Width Modulation) in conventional closed loop control are limited by phase current sampling and the execution of complex algorithms, especially in sensorless control algorithm will be lower. As far as the current microcontroller is concerned, the PWM carrier frequency is usually at dozens of kHz in closed loop. For the miniature hollow cup motor with minimum coil inductance, the short freewheeling time is hard for closed loop control system to improve the carrier frequency. Here is the dilemma: when the carrier period is too long and the current acquisition device in the closed loop controller is about to collect current and other feedback information, the freewheeling in inductance has been just over, then the whole closed loop control will not be conducted correctly.

On account of the fiber positioner robot is basically a constant torque load for the motor, and the inertia of 4 mm BLDC is small (0.00087 gcm$^2$), a super precision positioning control method of open loop control based on SVPWM (Space Vector Pulse Width Modulation) is proposed, which directly regulates the output electromagnetic torque of the motor through the corresponding strategy experiments, realizing the open loop positioning and placing the optical fiber to desired position precisely.

With regards to open loop positioning control, there is no need to collect feedback information (e.g. voltage, current), and run complex control algorithm in software. The carrier frequency of PWM can be easily raised to hundreds of kHz, which is very advantageous for miniature hollow cup motors with minimum coil inductance (e.g. the coil inductance of 4 mm motor we chosed is only 37 uH). Furthermore, robust positioning performance and strong reliability can be achieved simply. An open loop method is applied to the controller design of fiber positioner robot. And open loop driving parameters are regulated by experimental strategy and the specific effects of each parameter are discussed in detail. In order to assess the effectiveness of the positioning system, the positioning test is carried out on universal toolmaker’s microscope. The results reveals that the open loop positioning system satisfies the positioning requirements of the new generation LAMOST. And the hollow cup motor itself is more efficient than the stepper motor, reducing the heat dissipation of the whole system, creating a better thermal condition for the whole spectrum environment, decreasing the harmful effect of the positioning drive system to the seeing of astronomical observation.

## The open loop positioning control method

### SVPWM

**Figure 3:**
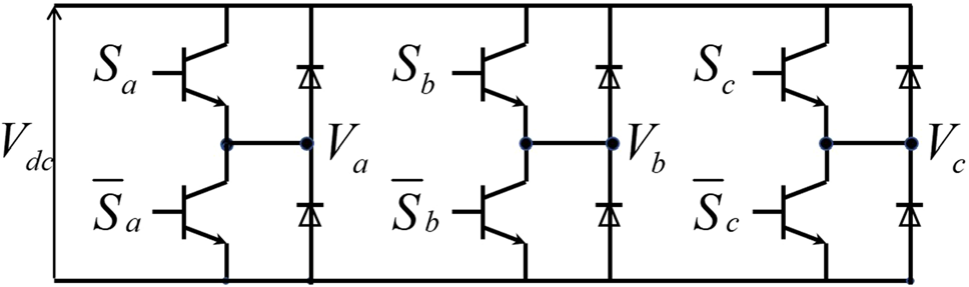
The hardware topology of three-phase two-stage H-bridge.

SVPWM^26^ is the most important part of digital vector control system. In voltage modulation, there is only one switch in each group of bridge arms (Fig. 3), and the output voltage is:

1 $$\begin{aligned} {V_x} = \left\{ {\begin{array}{*{20}{l}} {V_{dc}} & \qquad {\mathrm{{ }}{S_x} = 1}\\ {0} &\qquad {\mathrm{{ }} {S_x} = 0} \end{array}} \right. \end{aligned}$$

Let ${V_n} = ({V_a} + {V_b} + {V_c})/3$, the output voltage vector of the three-phase H-bridge can be expressed as:

2 $$\begin{aligned} \left[ {\begin{array}{*{20}{c}} {{V_{an}}}\\ {{V_{bn}}}\\ {{V_{cn}}} \end{array}} \right] = \frac{{{V_{dc}}}}{3}\left[ {\begin{array}{*{20}{c}} 2&{}{ - 1}&{}{ - 1}\\ { - 1}&{}2&{}{ - 1}\\ { - 1}&{}{ - 1}&{}2 \end{array}} \right] \left[ {\begin{array}{*{20}{c}} {{S_a}}\\ {{S_b}}\\ {{S_c}} \end{array}} \right] \end{aligned}$$

**Figure 4:**
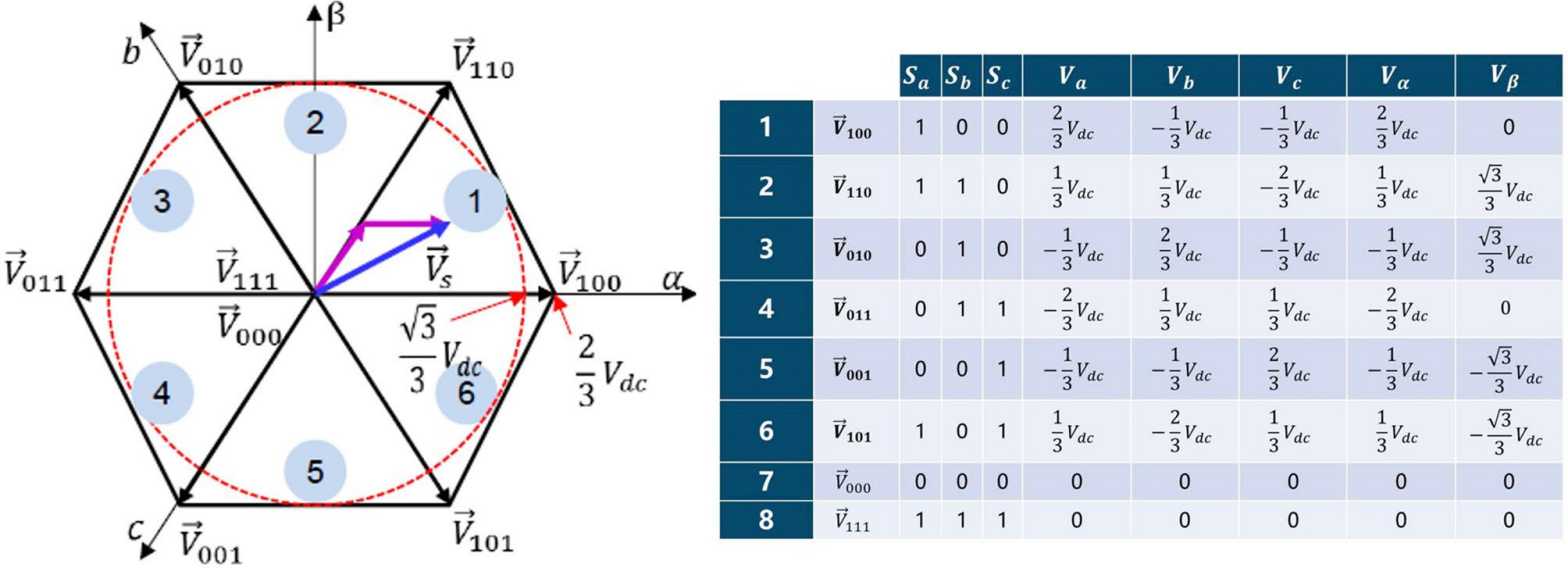
Vectors space distribution, $V_{dc}$ is the bus voltage.

There are 8 switching states of power devices in three-phase inverter, among which two outputs are “zero vector”, their amplitude is 0 without phase. The other six vectors space 60ř distribution, and the amplitude is $2/3 {V_{dc}}$ (Fig. 4).

### Space vector synthesis

**Figure 5:**
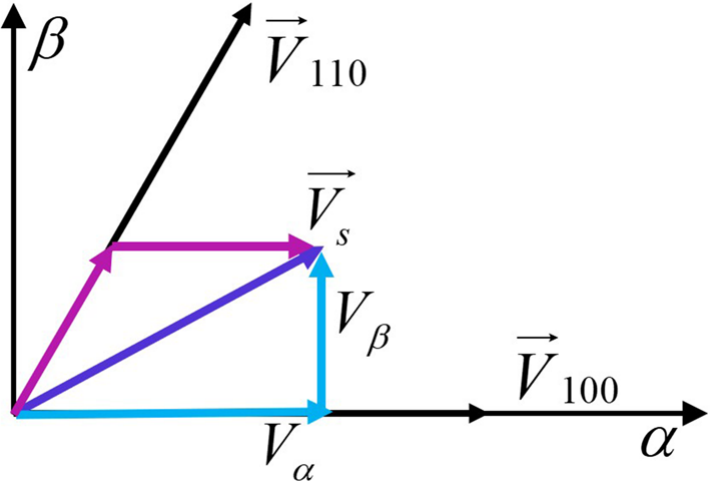
One sector of six.

For any space vector, it can be synthesized by adjusting the pulse width of the basic vector, the space vector $\overrightarrow{{V_s}}$ can be synthesized by the sum of the basic vector ${\overrightarrow{V} _{100}}$ and ${\overrightarrow{V} _{110}}$ (Fig. 5). When the carrier frequency is high enough, the synthesis of vectors can be derived by volt-second balance:

3 $$\begin{aligned} T\overrightarrow{{V_s}} = T\left[ {\begin{array}{*{20}{c}} {{V_\alpha }}\vspace{1ex}\\ {{V_\beta }} \end{array}} \right] = {T_1}{\overrightarrow{V} _{100}} + {T_2}{\overrightarrow{V} _{110}} = {T_1}\left[ {\begin{array}{*{20}{c}} {\displaystyle \frac{2}{3}}\vspace{1ex}\\ 0 \end{array}} \right] {V_{dc}} + {T_2}\left[ {\begin{array}{*{20}{c}} {\displaystyle \frac{1}{3}}\vspace{1ex}\\ {\displaystyle \frac{{\sqrt{3} }}{3}} \end{array}} \right] {V_{dc}} \end{aligned}$$

where *T* is carrier period, ${T_1}$ is occupation time of ${\overrightarrow{V} _{100}}$, and ${T_2}$ ${\overrightarrow{V} _{110}}$. Therefore, the occupation time of the basic vector is respectively:

4 $$\begin{aligned} \begin{array}{l} {T_1} = \displaystyle \frac{T}{{{V_{dc}}}} \cdot \left( \frac{3}{2}{V_\alpha } - \frac{{\sqrt{3} }}{2}{V_\beta }\right) \vspace{1ex} \\ {T_2} = \displaystyle \frac{T}{{{V_{dc}}}} \cdot \sqrt{3} {V_\beta } \end{array} \end{aligned}$$

### Open loop control model description

Assume that the hollow cup motor matches the following conditions:

- The rotor permanent magnet has no damping effect.
- Ignoring the spatial harmonics and assuming that the three-phase windings are symmetrical and the spatial difference is $120^\circ$, the magnetomotive force is distributed according to the sinusoidal law around the air gap.
- The magnetic permeability of rotor permanent magnet material equals 0, the same as that of air permeability.

**Figure 6:**
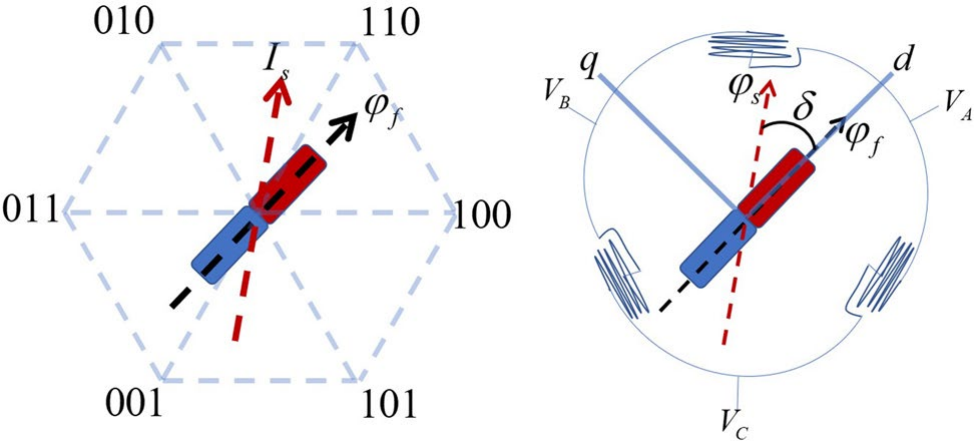
Stator and rotor of motor.

In open loop control model, firstly, we should deal with the problem of the motor rotor’s initial positioning. Here is the strategy, the MCU (Microcontroller Unit) read the vector information stored in the FLASH at the end of the previous positioning operation. I.e., after every time of optical fiber positioner robot finishing the positioning task, the last positioning vector executed will be written into the FLASH of the MCU for storage. When the next power-on run, the controller reads the last vector stored before from FLASH and takes it as the output of the initial positioning vector at current task, so that the rotor continues a new positioning and running from the position where it stopped last time.

After the initial positioning of the motor rotor, the stator current vector ${I_s}$ (Variables involved are displayed in Fig. 6.) in a specific rotation direction is applied, and the rotor can be brought into the open loop rotation from standstill. The voltage equation of the motor can be given by:

5 $$\begin{aligned} \begin{array}{l} {V_d} = r{I_d} + {L_d}\displaystyle \frac{{d{i_d}}}{{dt}} - {L_q}{I_q}{\omega _e}\vspace{1ex} \\ {V_q} = r{I_q} + {L_q}\displaystyle \frac{{d{i_q}}}{{dt}} + ({L_d}{I_d}+{\varphi _f}){\omega _e} \end{array} \end{aligned}$$

where, ${V_d , V_q}$—$d-$ and $q-$axis stator voltage, *r*—The motor phase resistance, ${I_d , I_q}$—$d-$ and $q-$axis armature current, ${L_d , L_q}$—$d-$ and $q-$axis inductance, ${\omega _e}$—Rotor electric angular speed, ${\varphi _f}$—Flux generated by rotor.

The three-phase coordinate system makes an equal amplitude clarke transformation to the $d-q$, so the power is reduced to 2/3 of the original value, in that case, the power consumed by the motor must be multiplied by 3/2, then the active power consumed by the motor is:

6 $$\begin{aligned} P = \frac{3}{2}({V_d}{I_d} + {V_q}{I_q}) \end{aligned}$$

According to equation (5), we can obtain

7 $$\begin{aligned} P = \underbrace{\frac{3}{2}r(I_d^2 + I_q^2)}_{\text{ winding } \text{ resisce } \text{ heating }} + \quad \mathrm{{ }}\underbrace{\frac{3}{2}\left[ {{\varphi _f} + ({L_d} - {L_q}){I_d}} \right] {I_q}{\omega _e}}_{\text{ output } \text{ mechanical } \text{ power } P_{mech}} \end{aligned}$$

The mechanical power of the motor is ${P_{mech}} = {\tau _e}{\omega _{mech}}$, where ${\tau _e}$ represents the electromagnetic torque. Based on electric frequency ${\omega _e} = p{\omega _{mech}}$ (*p* is the pole pairs), we have

8 $$\begin{aligned} {\tau _e}{\omega _{mech}} = \frac{3}{2}\left[ {{\varphi _f}+ ({L_d} - {L_q}){I_d}} \right] {I_q}p{\omega _{mech}} \end{aligned}$$

Then formula of electromagnetic torque can be given by:

9 $$\begin{aligned} {\tau _e} = \frac{3}{2}\left[ {{\varphi _f}\mathrm{{ + (}}{L_d} - {L_q}\mathrm{{)}}{I_d}} \right] p{I_q} \end{aligned}$$

In view of stator flux ${\varphi _s}$ and rotor flux ${\varphi _f}$, and the angle between them is called torque angle $\delta$, we can establish

10 $$\begin{aligned} \left\{ {\begin{array}{*{20}{c}} {{\varphi _d} = \left| {{\varphi _s}} \right| \cos \delta }\\ {{\varphi _q} = \left| {{\varphi _s}} \right| \sin \delta } \end{array}} \right. ,and \left\{ {\begin{array}{*{20}{c}} {{\varphi _d} = {L_d}{I_d} + {\varphi _f}}\\ {{\varphi _q} = {L_q}{I_q}} \end{array}} \right. \end{aligned}$$

The current equation in the $d-q$ coordinate system can be expressed by:

11 $$\begin{aligned} {I_d} = \frac{{\left| {{\varphi _s}} \right| \cos \delta - {\varphi _f}}}{{{L_d}}},{I_q} = \frac{{\left| {{\varphi _s}} \right| \sin \delta }}{{{L_q}}} \end{aligned}$$

Substituting the equation (11) into 9 yields^27^:

12 $$\begin{aligned} {\tau _e} = \frac{{3Pn}}{{Ld}}\left| {{\varphi _s}} \right| {\varphi _f}\sin \delta + \frac{{3(Ld - Lq)}}{{4LdLq}}{\left| {{\varphi _s}} \right| ^2}\sin 2\delta \end{aligned}$$

The first part in equation (12) is the electromagnetic torque, which is caused by the magnetic field interaction between the motor stator and the rotor, and the second part in equation (12) is the magnetoresistive torque, which is caused by the convex polarity of the motor. As for a micro hollow cup motor, the permanent magnet of the rotor is composed of a whole piece of magnet, which does not have convex polarity, therefore, the value of second part equals 0, and the electromagnetic torque equation of the motor is transferred into:

13 $$\begin{aligned} {\tau _e} = \frac{{3Pn}}{{Ld}}\left| {{\varphi _s}} \right| {\varphi _f}\sin \delta \end{aligned}$$

Considering a very short time $\Delta t$ when the motor starts, in open loop control, ${\varphi _f}$ follows ${\varphi _s}$ closely, since the inertia equaling 0.00087 ${\mathrm{gcm}}^2$ is relatively small. Moreover, we can assume that $\delta \approx \displaystyle \frac{{360^\circ }}{{subvision}}$, which can be considered as fixed, anyway. Where $\left| {{\varphi _s}} \right| = \int {({V_s} - r{I_s})dt}$ is the only variable we can regulate (${V_s}$ represents phase voltage). $\left| {{\varphi _s}} \right|$ can be further computed as

14 $$\begin{aligned} \left| {{\varphi _s}} \right| = \overline{{V_s}} \cdot \Delta t - r\overline{I} \cdot \Delta t \end{aligned}$$

where $\overline{I}$ is the AC (Alternating Current) average value and $\overline{{V_s}}$ the average value during the time of $\Delta t$, the parameters that can be regulated for the electromagnetic torque at the start-up stage are $\Delta t$ and $\overline{{V_s}}$.

## Open loop regulating process

**Figure 7:**
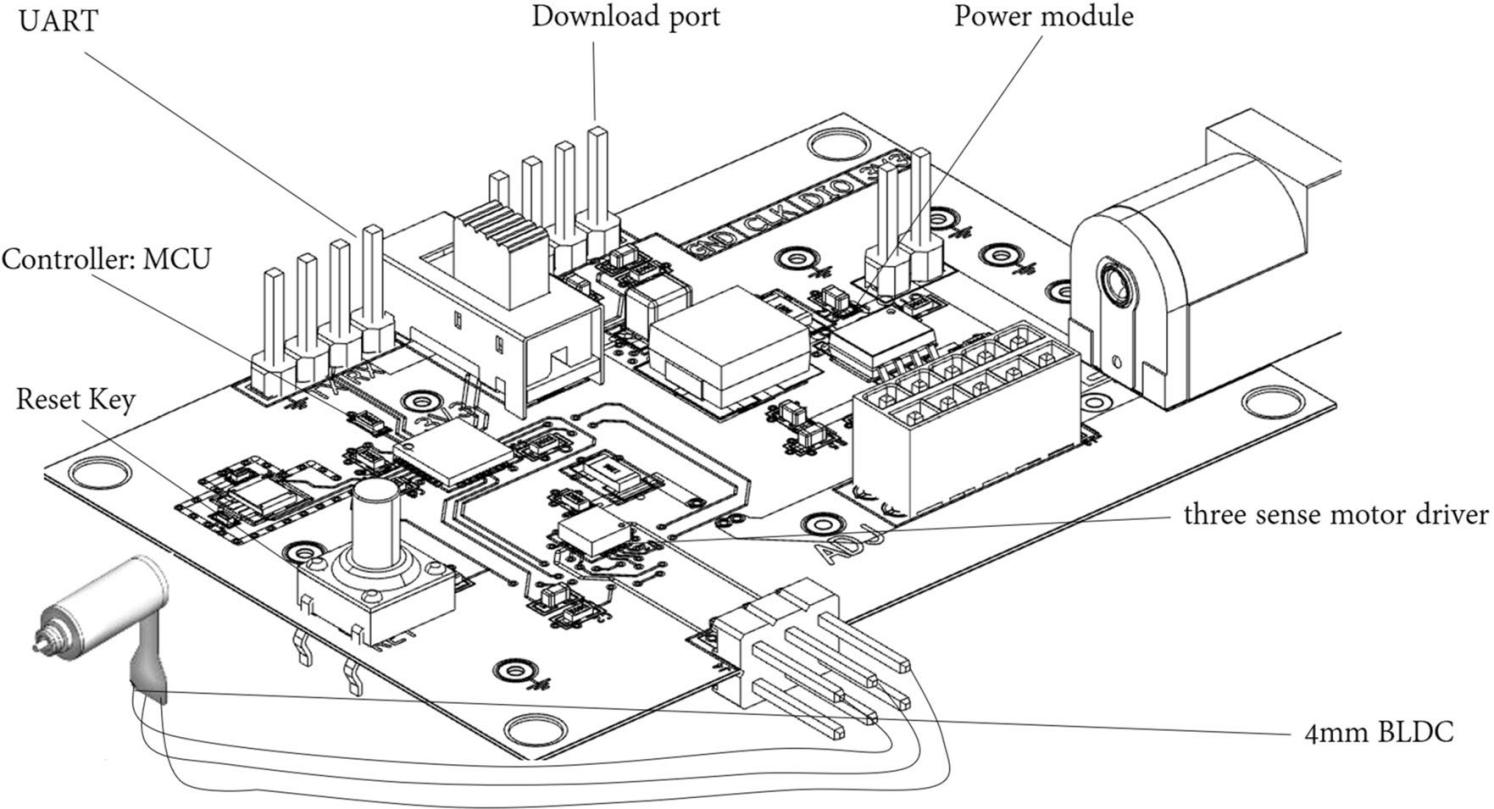
Printed circuit board designed for experiments.

The SVPWM module (Fig. 7) mainly consists of a MCU, whose timer TIM1 to generate three-phase SVPWM pulses, and another timer TIM2 to switch the vector table of TIM1 output, achieving the aim of motor commutation. The vector table is stored as an array in the FLASH; suppose there are *N* vectors in one turn of dragging motor, and after the TIM2 commutation *N* times, the magnetic field of the stator rotates one whole turn. In each TIM2’s count cycle of ${T_2}$, TIM1 outputs the same vector *n* times, in which *n* is determined by the experiment according to the specific load. Among them, the PWM fundamental frequency *f* is the reciprocal of TIM1’s base cycle, i.e., ${t_{base}}=1/f$, ${T_1}={t_{base}} \cdot {V_{counter1}}$, ${T_2} = n \cdot {T_1}$, where *n* is an integer, ${V_{counter1}}$ is TIM1 cycle count value; for a specific load torque, it can be known that when ${V_s}$ is constant, the larger *n* is, the greater the motor output torque will be, and the corresponding phase current increases. By conducting experiments, the value of *n* is continuously regulated to fullfil the task of successfully dragging the load of motor.

**Figure 8:**
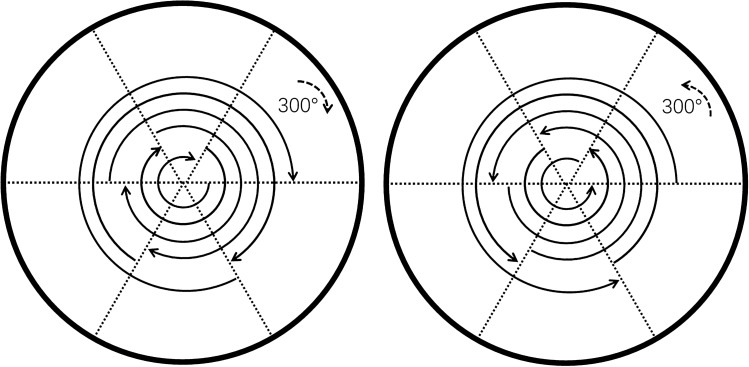
A back and forth movement of 300 degrees at the positioner to go in a row both clockwisely and anticlockwisely.

Considering the fluctuation of the load torque at different starting positions actually, the following rules are made about the definition of the motor dragging the load successfully (Fig. 8): commanding the positioner to spin $300^\circ$ for six times continuously in both positive and negative direction of motor, only if all the 12 operation of positioning are smoothly, the open loop drive parameter would be regarded as proper. Alternatively, if the open loop parameters are able to drag the load of fiber positioner robot, the driving torque should be larger than load torque of the positioner at any position.

If the load torque of the motor varies according to the starting position, the differential fluctuation is ${F_1}, {F_2}, {F_3}...$, you should make sure that driving torque ${T_e} > max\{{F_1}, {F_2}, {F_3}...\}$. By the means of Fig. 8, it is easily obtained that the critical number of times acting on the same vector under a certain bus voltage, and when $m >= n$, the corresponding starting torque satisfies: ${T_e} >= max\{{F_1}, {F_2}, {F_3}...\}$, so *n* is the critical value to be tested yeilding the break-away torque.

Since ${\overline{I}}$ is hard to measure, it could be qualitatively known that with the increase of voltage, the ${\overline{I}}$ increases, and the corresponding value of ${\overline{{V_s}} \cdot \Delta t}$ in 14 also increases equation. Let $L({V_s},t) = \overline{{V_s}} \cdot \Delta t = \left| {{\varphi _{s1}}} \right| + r\int {{I_s}dt}$, which is obviously a function of phase voltage ${V_s}$ and time *t*, where $\left| {{\varphi _{s1}}} \right|$ represents the load torque, which is constant for a fixed load; the phase voltage $\overline{V_s}$ in the formula is directly replaced by the bus voltage measured by voltmeter, ${V_{bus}} = \overline{{V_s}} \cdot a$, *a* is a constant coefficient and do not care its detailed value. The $\Delta {t}$ is directly replaced by $n \cdot {T_1}$, where ${T_1}$ is the count cycle of TIM1, *b* is a certain constant coefficient. Then $L({V_s},t)$ can be further computed as

15 $$\begin{aligned} L({V_s},t) = \overline{{V_s}} \cdot \Delta t = \frac{T_1}{a} \cdot {n}{V_{bus}} \end{aligned}$$

**Figure 9:**
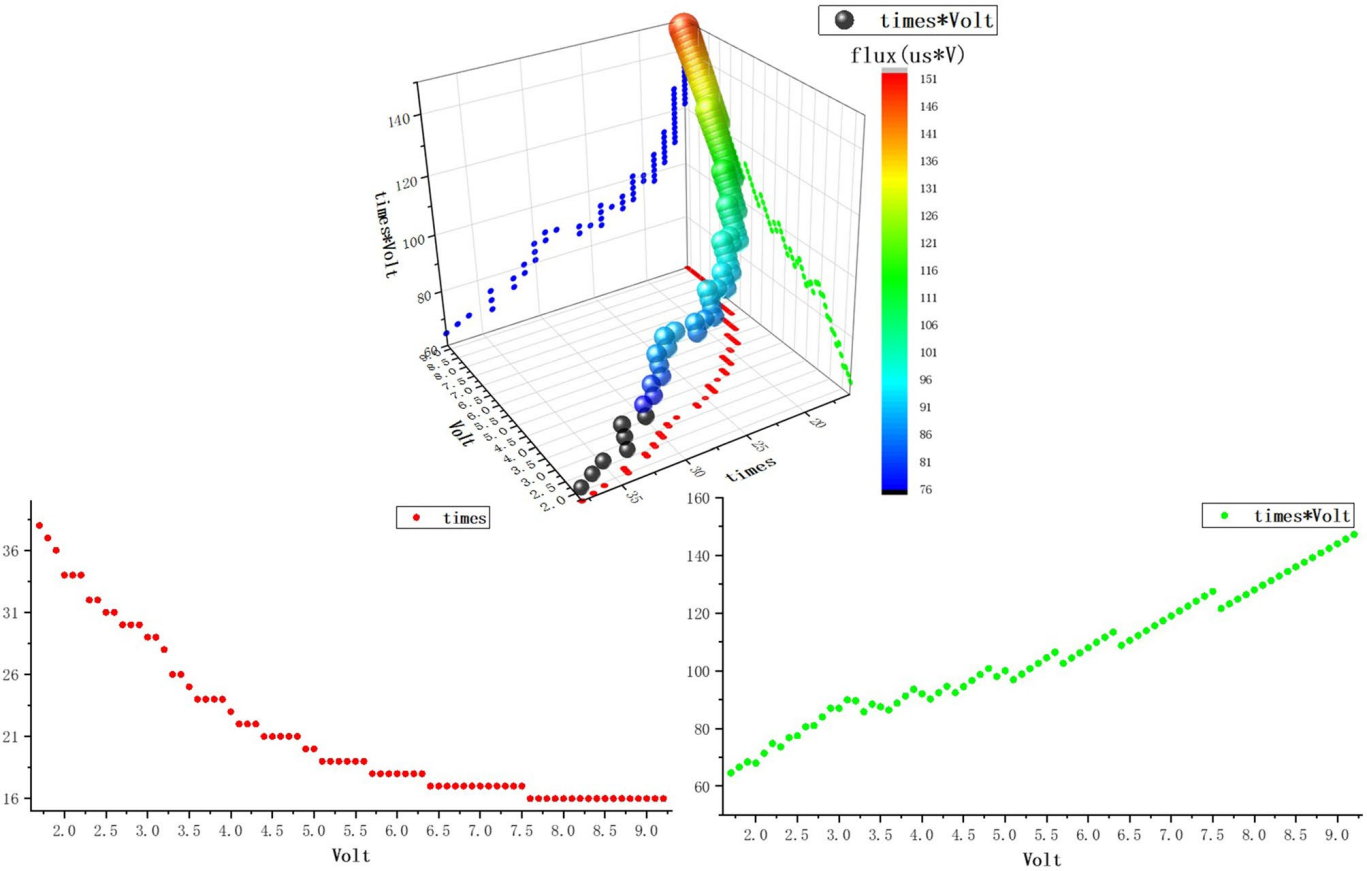
A three-dimensional graph reflecting the relationship among ${V_{bus}},n,{V_{bus}}\cdot n$.

Bying commanding the positioner to run as Fig. 8 discribes and then increasing *n* by degree till find out the reliable value of *n* under the $${V_{bus}}$$ varies from 1.7 Volt to 9.2. In this way, we check out a series of *n* as $${n_1}, {n_2}, {n_3}... $$ Draw three-dimensional graph with $${V_{bus}},n,{V_{bus}}\cdot n$$ (Fig. 9).

We can observe from the results (Fig. 9) that:

- As ${V_{bus}}$ increases, *n* decreases accordingly.
- When ${V_{bus}}$ increases, $L({V_s},t)$ representing flux increases gradually.

In the open loop driving strategy, the torque fluctuation is inevitably introduced. In view of this, it is discussed from the following aspects to optimize it: subdivision, PWM fundamental frequency, wave generation mode.

## Three aspects for optimization and Peak current

**Figure 10:**
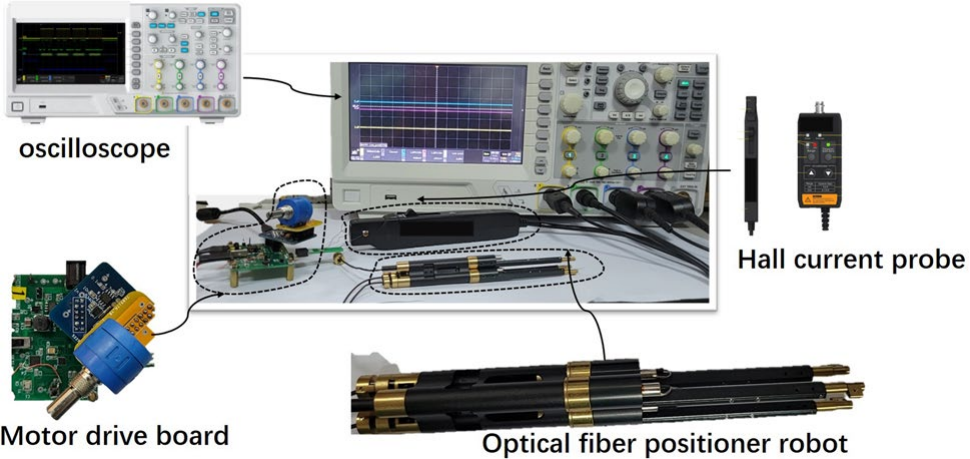
Experiment platform.

Experiment platform (Fig. 10): $${V_{bus}} = $$ 9 Volt; the oscilloscope ZDS3024 adopts unified setting: voltage channel 5.00 Volt beat div; current channel test uses ZCP30 Hall current probe in 200 mA/div; oscilloscope time base setting: 1.00 ms/div. (If there are no special hint later, above setting will be used all the time.)

### Subdivision

**Figure 11:**
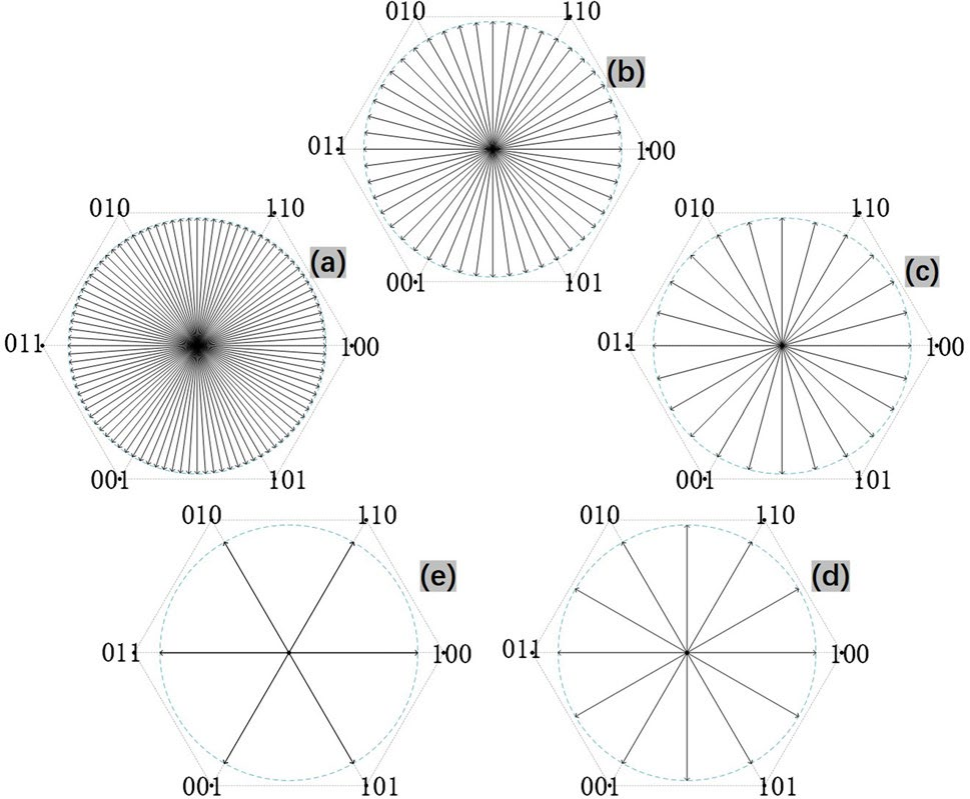
(**a**)$\sim$(**e**) correspond subdivision from 96 to 6 separately.

The experiment settings are as follows: the clock frequency of the generator TIM1 is 144 MHz, its cycle count value ${V_{counter1}}$ is 500, then the PWM fundamental frequency is 288 kHz (144 M /500=288 k). And the commutation TIM2 cycle count value ${V_{counter2}}$ changes according to the different subdivision (Fig. 11) to keep the same speed of stator magnetic field. The values of ${V_{counter2}}$ are shown Table. 2.

**Table 2:** Subdivision and ${V_{counter2}}$ (TIM2 cycle count value).

Subdivision	96	48	24	12	6
${V_{counter2}}$	8 $\cdot$ 500	16 $\cdot$ 500	32 $\cdot$ 500	64 $\cdot$ 500	128 $\cdot$ 500

**Figure 12:**
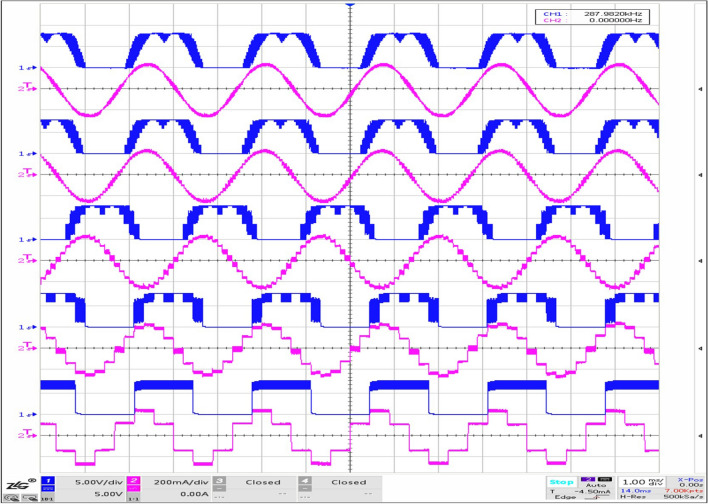
Experiment results of subdivision: from top to bottom displays the $I_s$ (red) and $V_a$ (blue) varying from subdivision 96 to 6 in turn.

The results from Fig. 12 show that

- With the increase of subdivision, the phase current $I_s$ is closer to the sine wave, and the line voltage $V_a$ is closer to the saddle wave modulated by SVPWM.
- When there are only six vectors in a rotation period, it is apparently that the phase current appears to be ladder-like of six steps, that is, the current pulsation will be stimulated at each vector commutation, causing the ripple of output torque. Therefore, the mechanical system would be not smooth enough, and noise will be greater. With no doubts, a larger subdivision is recommended in practical application. Based on this, we use 96 subdivision.

### Fundamental frequency

Keep the stator magnetic field speed same (adjust the TIM1 cycle count value ${V_{counter1}}$ and the commutation TIM2 cycle count value ${V_{counter2}}$), under the subdivision vector of 96:

**Table:** 

${V_{counter1}}$	2000	1000	500	250
${V_{counter2}}$	2000 $\cdot$ 2	1000 $\cdot$ 4	500 $\cdot$ 8	250 $\cdot$ 16

**Figure 13:**
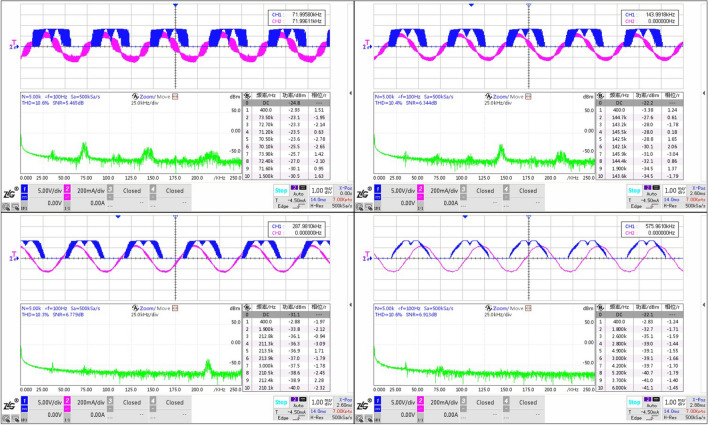
Experiment results of fundamental frequency: top-left for 72 kHz, top-right 144, bottom-left 288, bottom-right 576; red part for $I_s$, blue $V_a$, green frequency spectrum.

Record *SNR* (Signal-Noise Ratio) values as an evaluation indicator, reflecting the signal quality, defined as: $SNR = 10\lg (\displaystyle \frac{{{P_1}}}{{{P_{total}} - \sum \limits _{i = 1}^6 {{P_i}} }})$ (The oscilloscope regards the frequency point with the largest value except the DC component in the current spectrum as the signal, and the signal power in the formula is ${P_1}$, the harmonic power ${P_i}$, which removes the first six harmonics.).

**Table 3:** Fundamental frequency and SNR.

Fundamental frequency (kHz)	72	144	288	576
SNR	5.465	6.334	6.779	6.913

It can be seen clearly that the phase current ripple deceases with the increase of the fundamental frequency (Fig. 13), and this can also be supported by the frequency spectrum of the phase current (green part in Fig. 13). With the increase of the PWM frequency, the sine degree of the current waveform is better, which effectively reduces the torque fluctuation. Also from Table 3, we obtain that *SNR* reflecting signal quality would be better with the increase of fundamental frequency.

### Wave generation mode

**Figure 14:**
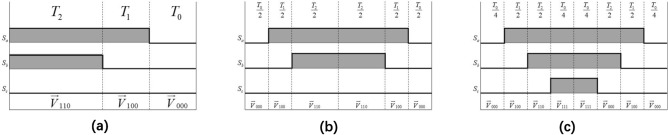
(**a**) stands for asymmetric wave, (**b**) five-segment symmetrical, (**c**) seven-segment symmetrical.

This section discusses the effect of asymmetric SVPWM, five-segment SVPWM and seven-segment SVPWM on the open loop positioning control proposed. SNR and AC RMS (Root Mean Square) of current are used for assessment. According to equation (4), there are three realizable approaches of SVPWM: asymmetric wave generation, five-segment symmetrical and seven-segment symmetrical (Fig. 14).

- (a) In asymmetric SVPWM, voltage vectors are synthesized by three basic vectors: ${\overrightarrow{V} _{000}} \rightarrow {\overrightarrow{V} _{100}} \rightarrow {\overrightarrow{V} _{110}}$. A carrier cycle contains two switch changes, with small switch loss and many voltage harmonics.
- (b) In a 5-segment SVPWM, the voltage vector is synthesized by three basic vectors: ${\overrightarrow{V} _{000}} \rightarrow {\overrightarrow{V} _{100}} \rightarrow {\overrightarrow{V} _{110}}$. A carrier cycle contains four switch changes, with moderate switch loss and moderate voltage harmonics.
- (c) In 7-segment SVPWM, voltage vectors are synthesized by four basic vectors: ${\overrightarrow{V} _{000}} \rightarrow {\overrightarrow{V} _{100}} \rightarrow {\overrightarrow{V} _{110}} \rightarrow {\overrightarrow{V} _{111}}$. A carrier cycle contains six switch changes, resulting in large switch loss and less voltage harmonics.

**Figure 15:**
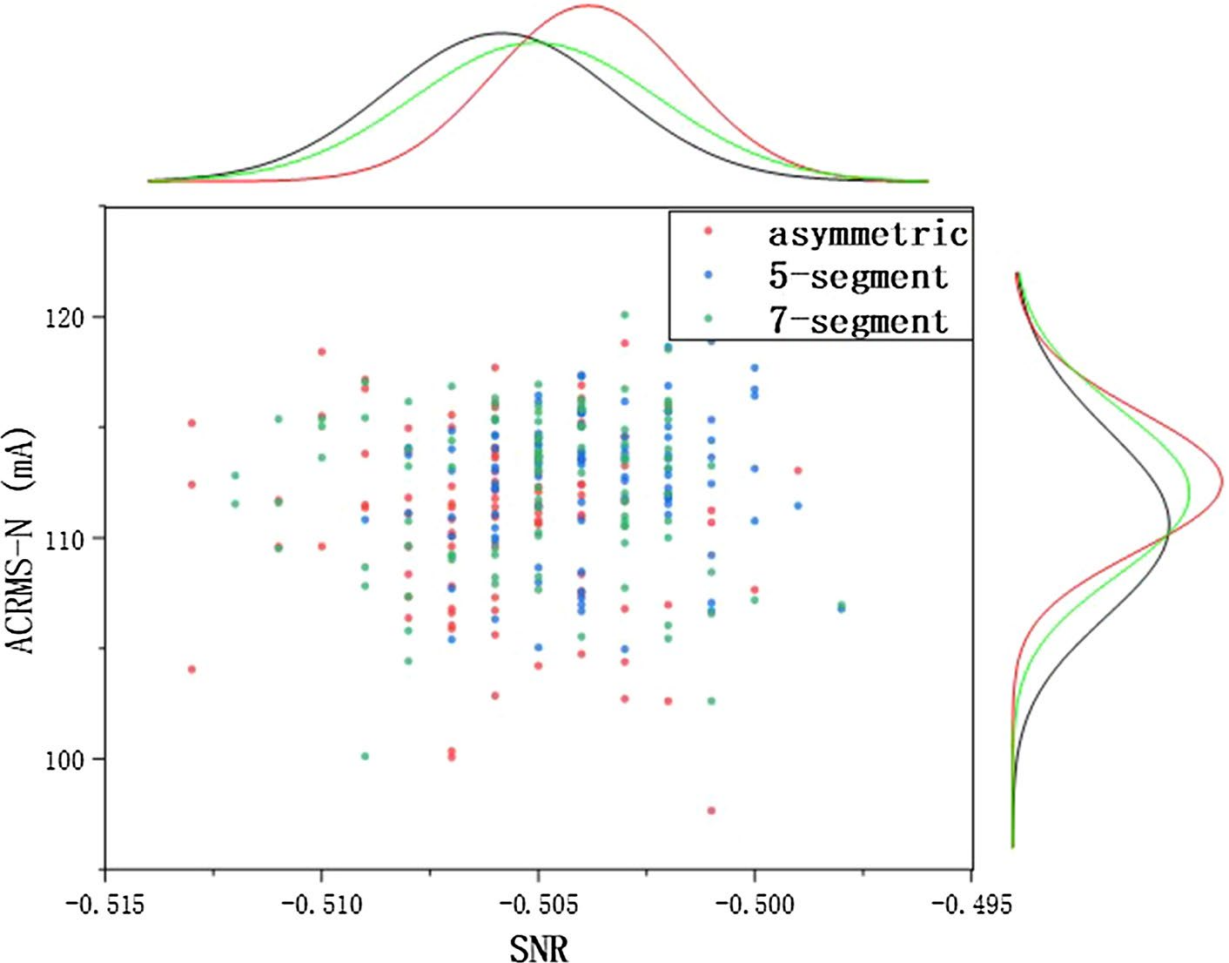
The marginal histograms.

For the above three modes of wave generation, under the same 9 *V* bus voltage, $V_{counter1}$ equals 500 and $V_{counter1}$ 4000, the marginal histograms are as Fig. 15 follows:

**Table 4:** SCRMS and SNR.

Wave mode	asymmetric	5-segment	7-segment
SNR	−0.5059	−0.5038	−0.5051
SCRMS (mA)	110.8562	112.6690	112.2300

From the Table 4, the harmonic difference is not obvious, but the current consumption is more relatively obvious, due to the reduction of switching times in one cycle of mosfet, the current loss is indeed smaller, while the current consumption of asymmetric generation is slightly smaller than that of the two other modes, but there is no significant difference between 5-segment and 7-segment. Because there will be 5000 optical fiber positioner robots in the new generation LAMOST positioning system, and every positioner robot is driven by two motors, although the current of asymmetric wave generation is only at least 1.37 mA less than that of the other two wave generation methods, but the power of one cycle is reduced by 1.37 ${\mathrm{mA}} \cdot$9 *V* =12.33 mw, multiplying by 10000, which will become a considerable amount. In that case, we tend to apply asymmetric wave generation mode.

### Key factors affecting peak current

**Figure 16:**
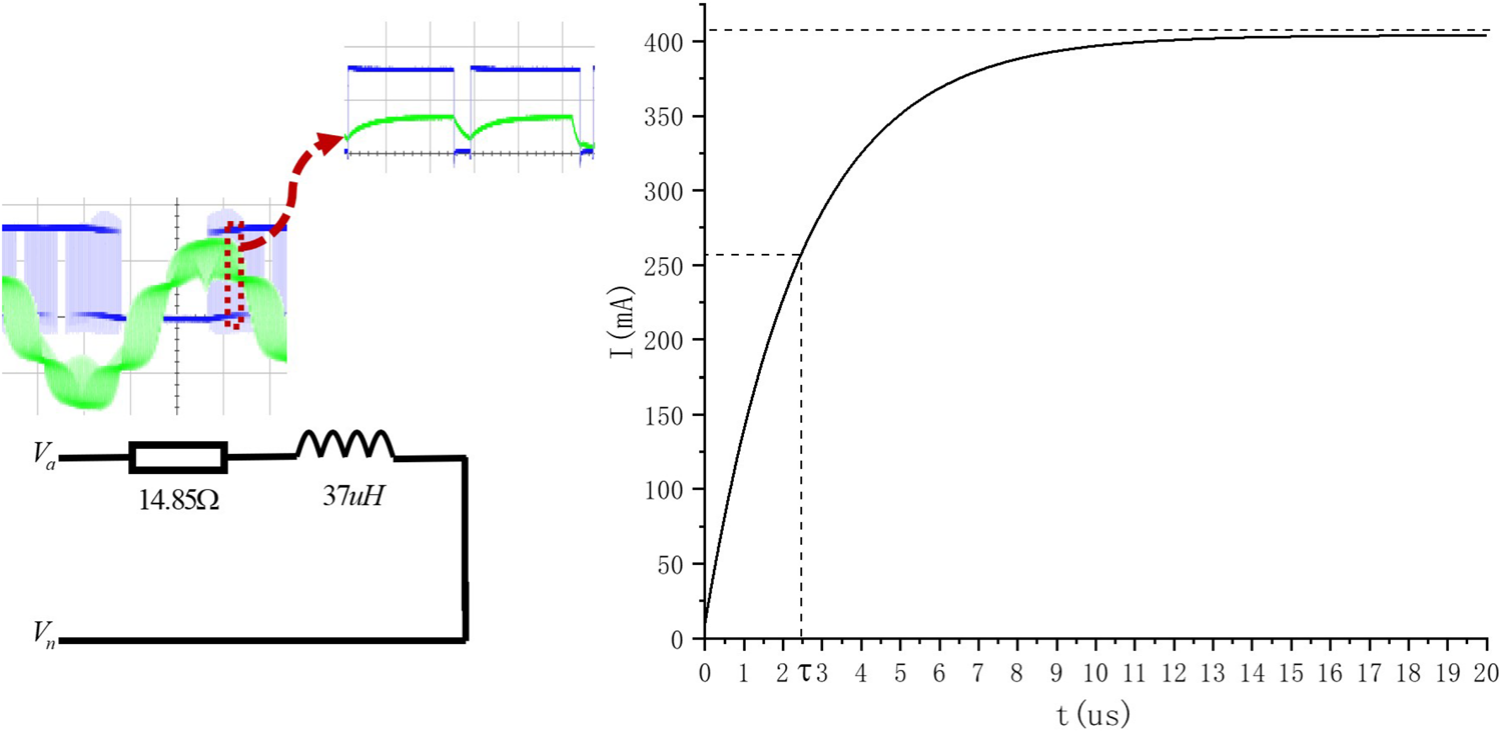
Single phase equivalent circuit of motor.

Analyze the single phase equivalent circuit shown in Fig. 16.

The full response expression of the first-order circuit in the Fig. 16 above is:

16 $$\begin{aligned} i(t) = i(\infty ) + [i({0_ + }) - i(\infty )]{e^{ - \frac{t}{\tau }}} \end{aligned}$$

where $i(\infty ) = \displaystyle \frac{{{V_a} - {V_n}}}{r}$, *r* is phase resistance of 14.85 ohm; $\tau$ is the electrical time constant of motor single-phase winding. $\tau = \displaystyle \frac{{{L_s}}}{r} =$ 2.49158 us.

**Figure 17:**
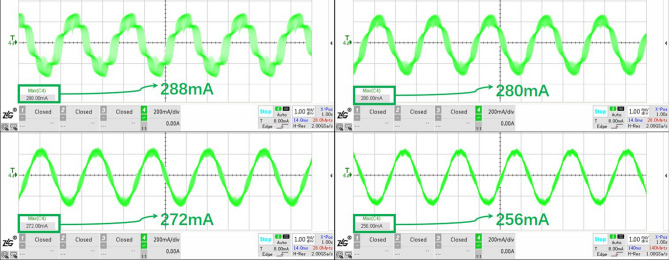
Experiment results of peak current: max high level counting value of TIM1 equaling ${V_{counter1}}$(top-left) is 2000, top-right 1000, bottom-left 500, bottom-right 250.

According to equation (16), we can conclude that the amplitude of the current fluctuation in the winding is related to the PWM high level time $t_{on}$. When $t_{on}$ increases, the current value of the above first-order circuit continues to rise. Considering the actual situation, when $t_{on}$ reaches a certain value, exceeding the allowable bandwidth of the hollow cup motor phase inductor, the windings will be saturated and the motor reaches the state of short-circuited, which must be seriously avoided. In that, the peak value of phase current measured by the current probe can be used to judge whether the regulated parameters are saturated or not, so as to eliminate the occurrence of dangerous situations. The clock of TIM1 is 144 *M* and ${t_{base}}$ equals 1/144 *us*. Considering the theoretical time required for the phase current to reach 0.632$\cdot i(\infty )$, the phase current is 0.632 times of the steady-state current when the high-level counting output value of 359. Based on experiments conducted in Sect. 4.2 Fundamental frequency, we test the peak current additionally. The results are shown in the Fig. 17 and Table 5.

**Table 5:** Peak current in various $t_{on}$.

Max high level counting value of TIM1	2000	1000	500	250
Peak current (mA)	288	280	272	256

It can be seen that with the increase of high level counting value, the peak value of phase current increases gradually. When the maximum high level of TIM1 is 2000, it has far exceeded 359, but in practice, the peak value of phase current is still only 288 mA, which does not reach the steady phase current 349.91 mA ($$9\ V / \sqrt{3} /14.85\ {\mathrm{ohm}}=349.91\ $$ mA). Due to the fact that the hollow cup motor windings has no iron core, and the magnetoresistance of the air is quite large, so the phase inductor is difficult to be saturated, of course, it does not mean that it will not be saturated, just that it has a large bandwidth for use. Hence, when regulate the open loop parameters, the reference value 359 can be calculated and checked in combination with the phase current peak value tested to prevent inductor saturation caused by improper open loop driving parameters. (This paper has an example in Sect. 5 about the process of checking).

## Positioning experiment

According to the experiments and analysis in Sect. 4, the final open loop parameters for optical fiber positioner robot are set as follows: 96 subdivision, asymmetric wave generation mode, and $V_{counter1}$ equals 500. Considering the stiffness of the mechanical system, the rotation speed should not be too fast, and there is a necessary margin for the driving torque, so $V_{counter2}$ must be greater than the critical value. The positioner spins a whole circle to be better at 10$\sim$40 s, meanwhile reducing the heat generation of the system, we determine the $V_{bus}$ to a very low value of 1.8 Volt.

In Sect. 3, the critical value of *n* corresponding to 1.8 V is 37; if we drag load directly with the critical value, considering that there are two reducers in machine design: 337 and 45/17 respectively, the time for positioner finishing $360^\circ$ can be calculated as: $(1/144\ M \cdot 500 \cdot 37 \cdot 96 \cdot 337 \cdot 45/17 = 11\ {\mathrm{s}})$.

**Figure 18:**
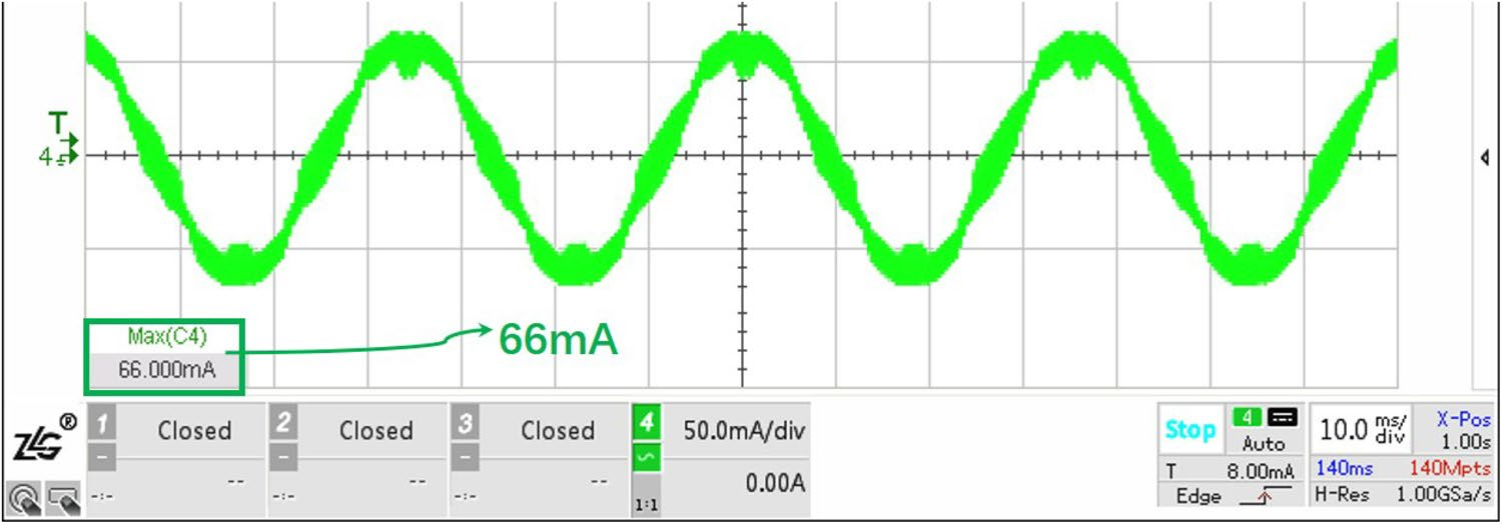
Peak current tested under $V_{bus}$ equaling 1.8 V.

Nevertheless, a slower speed and tougher stiffness requiring a certain margin of torque is desired. Finally, the $V_{counter2}$ is set to $500 \cdot 108$, so that the driving torque is nearly twice the margin, and time for positoner robot spining a circle will be about 32.1 *s*, guaranteeing a excellent stiffness. As for the problem of inductance saturation, it should be checked with the peak value of phase current tested (Fig. 18). The tested value of 66 mA does not reach the dangerous state of steady-state current equaling 69.98 mA ($1.8\ V \sqrt{3} /14.85\ \mathrm{ohm}$).

**Figure 19:**
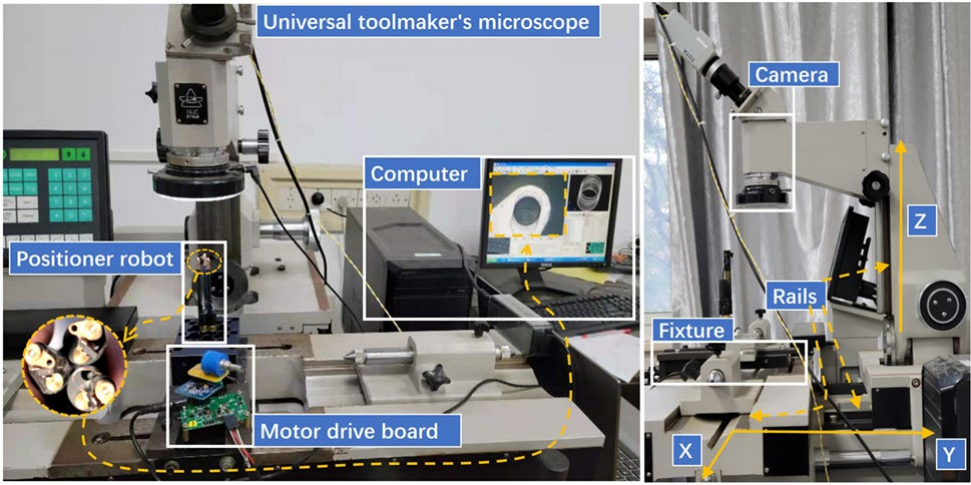
Positioning experiment platform.

The final positioning experiment is carried out on the universal toolmaker’s microscope (Fig. 19), which adopts close-range photogrammetry technology and uses the CCD (Charge Coupled Device) camera for precision position detection. Assuming face the camera, the movement of X-axis depends on worktable where the workpiece is clamped, in that the worktable can slide left and right freely; the adjustment of Y-axis relys on the worktable where the column is installed, which can move forward and backward; and the column component of microscope device responsible for load-bearing helps addressing the movement of Z-axis, so the camera can be easily lifted up and down in the direction of gravity. Through the precise motion of XYZ, the target markpoint of the positioner robot could be identified accurately. And in positioning experiment, the centre of the circle determined by the edge of the metal hole used to place the optical fiber in theta rotary axe is identified as the markpoint. The images obtained by camera are transferred into computer, then relevant software applys the image algorithms to extract the centre coordinates and record them.

The model of digital universal toolmaker’s microscope we operated is 19JC whose division value is 1 um in direction movement of X,Y and division value of Z-axis keeps 0.5 um. And the overall measurement accuracy is 1 um. Since the 19JC’s extent of automation and efficiency are not that high, manual measurement,adjustment and visual aiming are still necessary. The essential procedure of positionintg experiment are as follows:

- (a) Clamp the optical fiber positioner robot to the universal toolmaker’s microscope with the fixture.
- (b) Adjust sliding rails in the direction of XYZ, so that the markpoint can be obtained by camera.
- (c) The clockwise and counterclockwise alternating command is transmitted to the phi axe to spin $360^\circ$ every time.
- (d) After each positioning operation, the coordinate position of the markpoint is identified and recorded.

**Figure 20:**
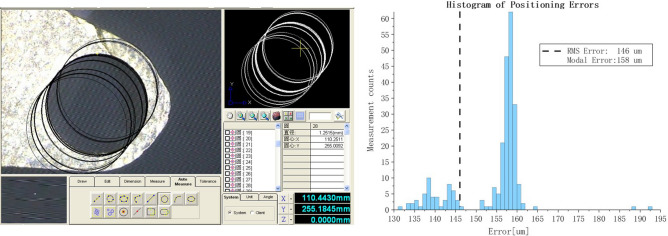
Positioning experiment results.

The clockwise and counterclockwise alternating positioning test is used to locate $360^\circ$ every time, and the results are recorded (Fig. 20).

The RMS value of the whole positioning error is 146 um, and the modal error is 158 um. Considering gear backlash, machenical and assembly error, and not all reliable mechanical connections used in the new generation positioner robot, especially for an adhesive means adopted, it can be concluded that the open loop positioning control proposed has achieved excellent positioning precision and meets the engineering demands.

## Conclusion

In this paper, an open loop precision positioning control method based on SVPWM is proposed, and the regulation process of every aspect and the specific influence involved are discussed in details. To some degree, the positioning control method could be improved furtherly with better IC (Integrated Circuit) selection, especially for maximum number of subdivisions and fundamental frequency. Take the MCU STM32F302K8U6 we used for example, the highest clock frequency of the generator timer is 144 Mhz, which is quite high. When adopting a carrier frequency of 576 kHz, its cycle count value $V_{counter1}$ is 250 ($144\ {\mathrm{Mhz}} / 576\ {\mathrm{kHz}} = 250$). At the same subdivision used, it is obvious that the higher carrier frequency is, the better the control appears. But the mosfet itself has rising, falling edges and integrated dead time, e.g. the mosfet we used whose rise time is 10 ns, fall 10 ns, dead zone 50 ns, when the timer’s base cycle ${t_{base}} = 1/f = 1/144 \ {\mathrm{Mhz}}= 6.94\ {\mathrm{ns}}$. In other words, the total time of the mosfet’s inherent drawback accounts for about 10 counts ($(10\ {\mathrm{ns}}+10\ {\mathrm{ns}}+50\ {\mathrm{ns}})/ 6.94\ {\mathrm{ns}}= 10$). If keeping the same subdivision, we insist on increasing fundamental frequency, the cycle count value $V_{counter1}$ must be reduced, then the time proportion of a PWM vector occupied by the the mosfet’s on-off will become larger, which obviously introduces uncertainties. As you can see, when using a fundamental frequency of 576 kHz (Fig. 13), $V_{counter1}$ equals 250, then the proportion of rise time, fall time and dead time adds up to 4% ($10/250 = 4\%$), which is not that low. So when increasing the carrier frequency, we have to consider the impact of the number of subdivision and the selected mosfet’s features, especially the rising, falling edges and dead time. Doubtlessly, the improvement of the carrier frequency for the hollow cup motor with minimum inductance is bound to realize a better sine phase current. However, the fiercely asending switch frequency of mosfet causing a higher junction temperature and leading to much more loss of electric energy can not be ignored. In that case, we finally decide to adopt 288 kHz fundamental frequency (the time wasted by mosfet deceases to 2% of single PWM cycle) and 96 subdivisions based on the existing IC selection, meanwhile, we have already noticed that the phase current appears a good sine degree and the motor is able to drag the load successfully.

As for the maximum number of subdivisions, when the number of subdivisions increases continuously with same carrier frequency, the more voltage vectors would be generated in same count cycle, and the high-level values of some voltage vectors in the vector table will decrease gradually to a certain extent, where the time wasted by mosfet would yield an assignable impact. Similarly, the maximum number of subdivisions is still subject to the inherent features of mosfet, here we no longer analyse unnecessary detail. If try a MCU with higher frequency timer and certain mosfet with more ideal on-off edges and shorter dead time, it is credible to achieve higher subdivisions and fundamental frequency.

Compared to the closed loop control limited by the current sampling and mathematical calculation of the feedback loop, the PWM carrier frequency of open loop method proposed is able to be as high as 576 kHz (Table 3), which is difficult to achieve in the closed loop control (even in the digital signal processors, the normal carrier frequency is dozens of kHz). If the MCU and mosfet permit, the open loop method can easily reach a higher carrier frequency even to MHz, which is especially beneficial for miniature hollow cup motors with short freewheeling time in minimum inductance. And the Fig. 13 shows that the open loop method proposed still ensures an excellent sine degree of phase current. Due to the torque fluctuation and thermal impact caused by the open loop strategy, some improvement approaches are put forward from fundamental frequency, subdivision and wave generation mode. Combined with the new generation of optical fiber positioner robot of LAMOST, a 1.8 V ultra-low voltage motor control system is designed of 4 mm miniature hollow cup motor with minimum inductance. The final positioning experiments are carried out on the universal toolmaker’s microscope, and the results validate that the positioner meets the stringent positioning requirements. The open loop method proposed is simple and robust with no sensors needed, what’s more, it has excellent practical value and engineering prospect in the field of precise positioning of miniature hollow cup motor especially for 4 mm. But inevitably, the energy utilization efficiency of the open loop control is lower than that of the closed loop.

## Prospect

In view of the open loop positioning control method proposed in this paper, there are still some optimation, a reasonable acceleration and deceleration curve in the starting stage under the premise of ensuring the robust output torque can further decease the heat loss. In addition, it is possible to develop an ultra-miniature 4 mm BLDC motor encoder to apply a closed loop positioning control in the future.
